# Patient-reported Outcome Measurements on the Tolerance of Magnetic Resonance Imaging-guided Radiation Therapy

**DOI:** 10.7759/cureus.2236

**Published:** 2018-02-27

**Authors:** Shyama Tetar, Anna Bruynzeel, Roosje Bakker, Marloes Jeulink, Ben J. Slotman, Swie Oei, Cornelis Haasbeek, Karel De Jong, Suresh Senan, Frank Lagerwaard

**Affiliations:** 1 Radiation Oncology, VU University Medical Center, Amsterdam, The Netherlands

**Keywords:** pro-q, mrgrt, stereotactic, adaptive, implementation, patient tolerance, smart

## Abstract

Purpose

Magnetic resonance imaging-guided radiation therapy (MRgRT) requires patient positioning within the MR bore and prolonged MR imaging during delivery, both of which are new in radiation oncology. Patient tolerance of MRgRT was prospectively evaluated using patient-reported outcome questionnaires (PRO-Q).

Methods

Our MRgRT procedure involves daily high-resolution MR scanning, limited re-contouring, daily plan re-optimization, quality assurance (QA), and gated delivery. Patients with claustrophobia are excluded. Mean fraction duration was 45 and 60 minutes for stereotactic treatments during free-breathing and breath-hold, respectively. Patient-controlled video-feedback was used for breath-hold delivery. PRO-Qs collected in the first 150 patients treated included questions on MR-related complaints and also evaluated aspects of active participation.

Results

Almost one-third of patients (29%) scored at least one PRO-Q item on MR-related complaints as ‘moderate’ or ‘very much’, with noise, feeling cold, and paresthesia being the most frequently scored in this way. Considerable anxiety was reported by 5%, but no medication was required for this in any patient. Patient participation in video feedback for breath-hold delivery was appreciated by the majority of patients, all of whom completed the procedure. Only 5% of patients considered treatment duration to be unacceptably long.

Conclusion

Despite the lengthy MRgRT procedure, outcomes of PRO-Q indicate that it was well-tolerated by patients.

## Introduction

A major recent advancement in radiation oncology is the implementation of magnetic resonance imaging-guided radiation therapy (MRgRT), which is now available in several centers worldwide. MRgRT offers several potential advantages, which can be used separately or in combination, including MR-based soft tissue setup, online MR imaging during delivery, markerless gated delivery, and daily adaptive radiotherapy [1]. In May 2016, MRgRT was introduced at our center for stereotactic MR-guided adaptive radiation therapy (SMART) using the MRIdian® system (ViewRay, Inc., Mountain View, CA), which combines a split 0.35 Tesla (T) MR scanner with ^60^Co radiation therapy [2].

Investigations to identify patient groups and indications which benefit from the use of MRgRT combined with daily plan adaptation are ongoing. Our institutional approach focused on indications where we expected a benefit from MRgRT combined with daily plan adaptation for each fraction. However, this approach leads to patients being positioned within the MR bore for a prolonged period of time. Such patients may experience procedure-related problems, such as anxiety, excessive noise, sensations of heat, and other MR-related complaints. Several studies have highlighted the importance of incorporating patient-reported outcome (PRO) measurements into routine clinical care [3-5]. PROs can be used to assess not only quality of life, but also patient experience and tolerance of treatment. Because of the novelty of MRgRT, we developed and prospectively collected PRO-questionnaires (PRO-Q) in patients undergoing this treatment. These PRO-Qs were used to evaluate patient tolerance, as well as to identify and improve aspects of our clinical MRgRT practice.

## Materials and methods

Description of the SMART procedure

**Figure 1:**
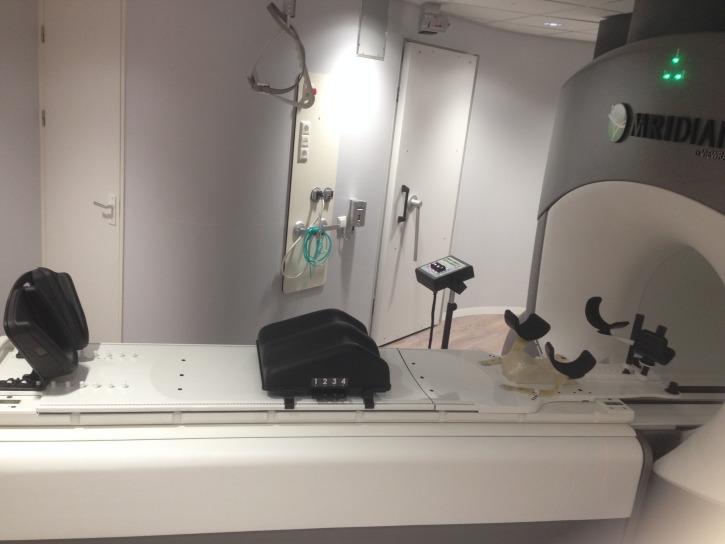
Custom-made MR-compatible positioning board, including foot, knee support, and arm support MR: magnetic resonance

At the initial consultation, information on the MRgRT procedure is provided, followed by CT simulation with use of dummy MR coils. Claustrophobic patients are initially identified by means of a pre-simulation MR-safety questionnaire. Subsequently, a simulation high-resolution (HR) MR-scan, without intravenous (IV) contrast, is performed on the MRIdian 0.35T machine. The duration of HR MR-scans ranges from 17 seconds (when performed at a shallow-inspiration breath-hold) to approximately three minutes for pelvic simulation scans. Positioning is performed on an MR-compatible positioning board (Macromedics, Waddinxveen, The Netherlands), including foot, knee, and arm support (Figure 1). As is customary in diagnostic MR-scanning, noise-reduction headphones are provided. For breath-hold delivery, patients were instructed during their first outpatient clinic visit in video feedback-assisted treatment delivery using anonymized case movies of previously treated patients. When tolerated by patients, those with upper abdominal and thoracic lesions are positioned with their arms up.

SMART treatment delivery

**Figure 2:**
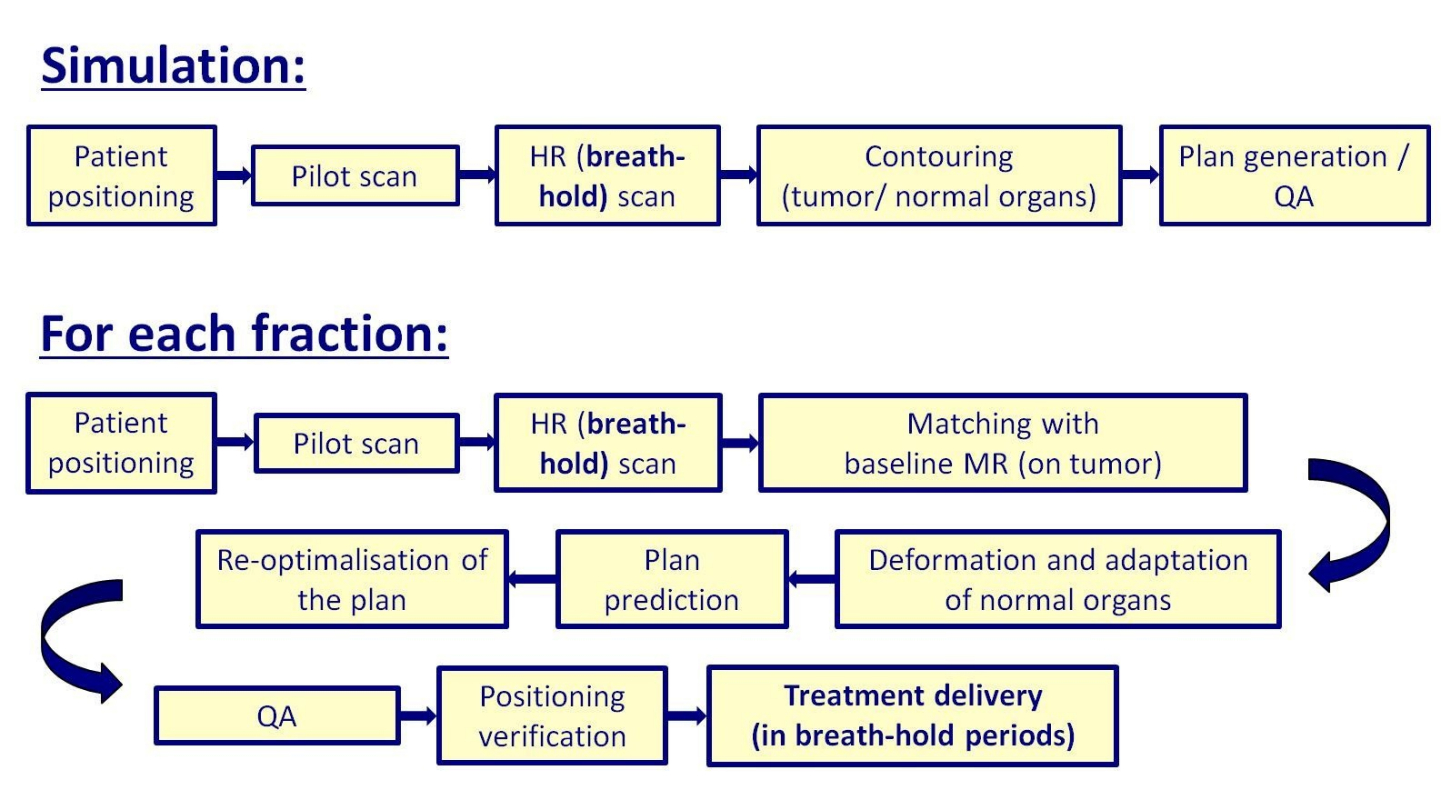
Clinical workflow for performing SMART at VUMC HR scan: high-resolution magnetic resonance scan; QA: quality assurance; MR: magnetic resonance; SMART: stereotactic magnetic resonance-guided adaptive radiation therapy; VUMC: Vrije Universiteit Medical Center

SMART delivery starts with positioning of the patient and MR coils, followed by a first rapid pilot scan (15 sec) to roughly align the patient. A repeat high resolution (HR) MR scan is then performed prior to each fraction and used for the final alignment of the gross target volume (GTV) or clinical target volume (CTV). Next, the contours of the organs at risk (OAR) are automatically deformed and manually adjusted by the clinician within a 3 cm distance from the planning target volume (PTV) [6]. Subsequently, the baseline intensity-modulated radiotherapy (IMRT) plan is re-calculated on the ‘anatomy of the day’. Plans are always re-optimized using the same number of beams and direction as the baseline plan but taking into account the current anatomy. After brief patient-specific quality assurance (QA), a single sagittal plane is selected for online tumor tracking and gated treatment delivery. The gating target is the contour that is tracked in each frame and is usually the GTV. The gating boundary is the gating target with a 3 mm margin added, and this is generally the PTV. During the above SMART steps, the patient remains in the treatment position, and radiation delivery is continuously monitored with MR-guidance. The clinical SMART workflow used at our center is depicted in Figure 2.

Breath-hold delivery

**Figure 3:**
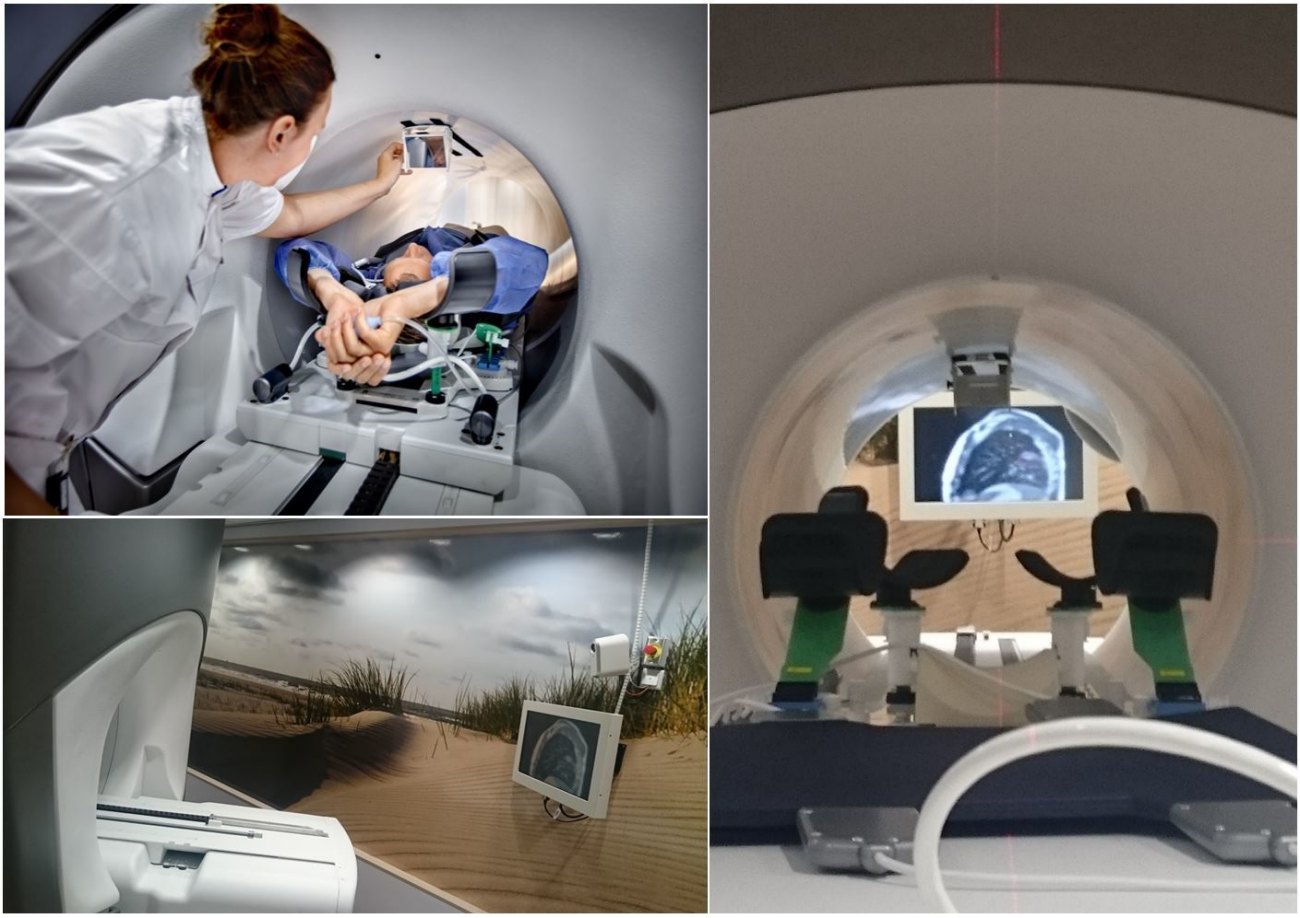
Illustration of video feedback system for breath-hold delivery Photograph: DigiDaan

MRgRT allows for a continuous monitoring of the target volume and other soft tissues during treatment delivery. On the MRIdian, a sagittal plane is imaged continuously at a frame rate of 4 per second. The gating target is projected on the sagittal plane and the gating boundary is used for gated delivery. When the gating target moves outside the pre-specified gating boundary, the radiation beam automatically stops. Using an in-house solution for video feedback to patients, patients can see the sagittal plane with the colored gating target and the gating boundary contours on an MR-compatible monitor (Cambridge Research) that is mounted on the wall at the head end of the bore. In this manner, patients have an active role (and at their own pace) in gated radiation delivery by keeping subsequent breath-holds using a mirror in the bore (Figure 3), which is also illustrated in Video 1 for three different patients with pancreatic cancer.

**Video 1:** Video Feedback System for MR-guided Radiotherapy Using Breath-hold Gated Treatment Delivery in Pancreatic Cancer Patients This video shows three examples of what patients actually see during their treatment. They observe in real-time the gating target (in blue or green) within the gating boundary (in red) on a sagittal tracking image derived from the MRIdian console. MR: magnetic resonance

Design of the PRO-Q

For assessing patient experiences, we developed an in-house PRO-Q including questions on potential MR-related complaints and experiences, such as anxiety, temperature, and noise. Patients were also queried on their general tolerance of the duration of the SMART procedure. In patients undergoing video feedback-assisted treatment delivery, specific questions on the element of active participation in their own treatment were assessed. Items could be scored on a 4-point scale as: “not at all”, “a little”, “moderate”, and “very much” (Table 1). In order to describe relevant problems encountered by patients, the scores “moderate” and “very much” for any question were combined and denominated “considerable” in this manuscript. PRO-Qs were collected once immediately following the last SMART fraction, and completion of the PRO-Q took five minutes on average. Statistical analysis was performed in Statistical Package for Social Sciences (SPSS), v.14 (IBM SPSS Statistics, Armonk, NY). Comparing mean values was performed using the analysis of variance (ANOVA) test.

**Table 1:** PRO-Q Used for This Study MRI: magnetic resonance imaging

During treatment	Not at all	A little	Moderate	Very much
Were you anxious inside the MRI bore?				
Was the treatment duration time acceptable?				
Inside the MRIdian, were you troubled by:	Not at all	A little	Moderate	Very much
Sensation of local heat?				
Feeling cold?				
Dizziness?				
Tingling or numb extremities				
A metallic taste?				
Perceptions of light flashes?				
Noise?				
Active role during delivery:	Not at all	A little	Moderate	Very much
Was it difficult to control the target by holding your breath?				
Was it confronting to see your tumor during treatment?				
Did you like having an active role during treatment?				
Did you worry about your contribution to the treatment?				

## Results

Between May 2016 to August 2017, we prospectively collected PRO-Qs in 150 patients treated with SMART. Included were 36 females (24%) and 114 males (76%) with a median age of 69 years (range: 35-92 years). Tumor sites treated are shown in Table 2, with prostate being the most frequent in 45%. The majority of patients were treated using a five-fraction stereotactic scheme, with the exception of several lung and liver lesions that received eight to 12 fractions.

**Table 2:** Treatment Characteristics (n = 150) n: number; SMART: stereotactic magnetic resonance-guided adaptive radiation therapy

Indication SMART	Number of patients	Percentage
Prostate cancer	68	45.3%
Pancreatic cancer	25	16.7%
Lung cancer	14	9.3%
Adrenal metastases	14	9.3%
Liver metastases	13	8.7%
Kidney cancer	9	6.0%
Other	7	4.7%
SMART delivery		
Breath-hold	80	53.3%
Free-breathing	70	46.7%

Anxiety during SMART

Despite initial screening using an MR safety questionnaire, two patients experienced severe claustrophobia during the simulation MR, resulting in their withdrawal from SMART treatment. Both were excluded from this analysis. Some degree of anxiety during SMART delivery was reported by 25 patients (17%), with seven of these patients (5%) reporting anxiety to be considerable. None of the patients needed medication for anxiety. Anxiety was reported significantly more frequently in women than men (31% vs. 12%; p = 0.01) and was not correlated with age (p = 0.38) nor the type of delivery (free-breathing or breath-hold (p = 0.24)).

Potential MR-related complaints

Eighty percent of patients reported at least some degree of one of the seven scored potential MR-related complaints. However, only 29% (N = 44) scored at least one of the former as being considerable. Despite the routine use of headphones, the disturbing noise was the most commonly reported complaint in 60% of patients, of whom 17% scored this as being considerable. Sensations of feeling cold during treatment were reported in 29% of patients, more frequently by women (p = 0.007) and by patients performing breath-hold delivery (p = 0.016). A similar percentage of patients experienced paresthesia, which was scored as considerable by 6%, and which significantly correlated with breath-hold delivery (p = 0.027). Other complaints, such as dizziness, local heat sensations, metallic taste, or light flashes, were only occasionally reported (Table 3).

**Table 3:** MR-related Complaints MR: magnetic resonance; N: number

	Yes	Considerable
Noise	60% (N = 90)	17% (N=26)
Cold	29% (N = 44)	10% (N = 15)
Paresthesia	28% (N = 42)	6% (N = 9)
Dizziness	11% (N = 16)	1% (N = 2)
Local heat sensations	9% (N = 13)	1% (N = 2)
Metallic taste	2% (N = 3)	-
Light flashes	2% (N = 3)	-

Patient experiences with the video feedback system

The sub-section of the questionnaire relating to patient-controlled breath-hold delivery using video feedback was completed by a total of 80 patients. Only 10 patients (12.5%) reported considerable difficulty to control the target position during breath-hold delivery. Their active role was appreciated by the vast majority (76%) of patients, with only eight patients (10%) answering that they would have preferred less active participation. Despite specific pre-treatment reassurance of patients that beam-off was automated when the tumor would be outside the gating window, seven patients (8%) remained concerned about their own contribution to treatment. All of these patients also scored the procedure as difficult. Finally, only three patients (4%) answered that seeing their own tumor during treatment delivery was considerably confronting (Table 4).

**Table 4:** PRO-Q Results Regarding the Video-feedback System (N = 80 patients) N: number

	Not at all	A little	Moderate	Very much
Was it difficult to control the target by holding your breath?	42% (N = 34)	45% (N = 36)	9% (N = 7)	4% (N = 3)
Was it confronting to see your tumor during treatment? (N=79)	86% (N = 69)	9% (N = 7)	3% (N = 2)	1% (N = 1)
Did you like having an active role during treatment? (N=79)	10% (N = 8)	13% (N = 10)	40% (N = 32)	36% (N = 29)
Did you worry about your contribution to the treatment?	62% (N = 50%)	30% (N = 23)	7% (N = 6)	1% (N = 1)

Tolerance of treatment duration

The mean duration of a single fraction was 45 minutes (range: 35 - 55 minutes) for free-breathing, and 60 minutes (range: 50 - 75 minutes) for breath-hold delivery, which includes all SMART steps, including positioning. Despite this prolonged duration, this was considered to be unacceptably long by only eight patients (5%), of which five were breath-hold patients.

## Discussion

Following its introduction in 2014, the use of MRgRT delivery, in combination with daily re-optimization of treatment plans, is expected to significantly expand in the near future. As patients are subjected to prolonged MR imaging during delivery, it is important to understand technique-related problems. To the best of our knowledge, ours is the first study evaluating the tolerance and problems encountered by patients during MRgRT. We used PRO-Q’s which are a proven tool in routine clinical care for understanding patient experiences, enhancing symptom management, and improving outcomes [5]. An in-house developed PRO-Q was used to collect experiences and potential MR-related complaints immediately after the last treatment fraction.

Despite an initial screening for severe claustrophobia, two patients declined MRgRT for this reason after the simulation step. Furthermore, some grade of anxiety was reported by 17% of our patients, more often in women. Our PRO-Q did not differentiate between claustrophobia and other potential causes of treatment-related anxiety, and this might be a future refinement of the questionnaire. These findings underscore the importance of providing pre-treatment information in the form of folders, video material on the departmental website, and experiencing the bore of the MRIdian during simulation. Patients report that regular audio communication with radiation therapists during treatment (informing them on progress and reassuring them) was very helpful. Patient feedback indicated that the use of a mirror inside the MR bore not only allows the patient to see the in-room monitor but also expands the view of patients during treatment, and this simple measure decreases mild claustrophobia.  

Despite routinely using noise-reduction headphones with optional music of choice, the disturbing noise was most the commonly reported MR-related complaint in 60% of patients, 17% of whom scored it as being considerable. For the MRIdian, this noise is not only caused by the MRI itself but also from retraction of cobalt sources, e.g., prior to the machine rotating for subsequent beam groups. This noise is expected to be less with newer MR-linac machines. Patients felt cold due to the cooling airflow of the machine; this was experienced mainly by breath-hold patients, which, on average, can take up to one hour. In the meantime, the temperature of the cooling air has been adjusted for patient comfort.

The SMART workflow at our center includes daily re-optimization of treatment plans, and the total duration of a single fraction was 45 minutes on average and one hour for free-breathing and breath-hold delivery, respectively. All upper abdominal and thoracic lesions have been treated using this breath-hold technique. Currently, about one-third of the in-room duration is taken by dose delivery, which will be restricted when MR linacs are used. However, MRgRT with daily adaptive radiotherapy will remain more time-consuming than conventional linac treatments. In general, this prolonged delivery was well tolerated, with only 5% of patients reporting that this was unacceptably long.

As treatment delivery on the MRIdian is co-planar, patient positioning with the arms above the head using the MR-compatible positioning board is preferred, particularly for breath-hold targets in the upper abdomen and thorax. However, paresthesia during the lengthy delivery was commonly reported, and on occasion, the simulation had to be repeated with either one or both arms down the side. As such, this appears more as positioning and treatment duration-related than caused by the magnetic field. Currently, patients who are treated with breath-hold are often scanned with arm(s) down, with planning beams avoiding going through the arms. Other potential MR-related complaints, such as vertigo, sensations of light flashes, and metallic taste, were only sporadically reported and always mild in nature. Despite the prolonged MR imaging, local heat sensations were uncommon, although this may be due to the relatively low magnetic strength of 0.35 T of the MRIdian.

We also addressed the video feedback system in the PRO-Q. Despite initial concerns, the majority of patients appreciated the active participation in their treatment. Video-assisted breath-hold appeared to be feasible, even in elderly patients, occasionally supported with audio feedback. This video-feedback can be performed with the help of the patient’s own glasses, if MR-compatible, or using adjustable MR-safe spectacles. Despite reassurance that beam-off was automated when the tumor would be outside the gating window, several patients remained concerned about their own contribution to the treatment. However, only three patients indicated that they found it confronting seeing their own tumor during treatment delivery. About one in eight patients reported this procedure as being considerably difficult; however, all patients were able to complete the treatment. Although difficult to derive from the current data, we generally observed a learning curve both during delivery and in between subsequent fractions.

Some limitations of our study deserve to be mentioned. We are fully aware that our in-house-developed PRO-Q may be subject to patients' own interpretations and could be biased by background and desirability of answers [7-8]. Our results may be influenced by the relatively low magnetic field of 0.35 T of the MRIdian and may not be extrapolated to future MR linacs with higher field strengths. Finally, our MRgRT approach was prolonged, not only because we treat with a high dose per fraction, but also because all fractions are performed with daily adaptation, i.e., re-optimizing baseline treatment plans for each fraction. This will certainly have an impact on patient tolerance.

## Conclusions

In summary, this evaluation of PRO-Qs shows that MRgRT, combined with daily adaptation, is feasible and generally well tolerated by patients. Our experience in the first year has allowed us to refine our workflow in order to decrease patient discomfort. Finally, the use of video feedback to patients may set a standard for future MR-guided delivery.
